# Correlation between Polycyclic Aromatic Hydrocarbons in Wharf Roach (*Ligia* spp.) and Environmental Components of the Intertidal and Supralittoral Zone along the Japanese Coast

**DOI:** 10.3390/ijerph18020630

**Published:** 2021-01-13

**Authors:** Masato Honda, Koki Mukai, Edward Nagato, Seiichi Uno, Yuji Oshima

**Affiliations:** 1 Botanical Garden, Institute of Nature and Environmental Technology, Kanazawa University, Kakuma, Kanazawa, Ishikawa 920-1192, Japan; 2 Faculty of Agriculture, Kyushu University, 744 Motooka, Nishi-ku, Fukuoka 819-0395, Japan; x610155k@gmail.com (K.M.); oshima.yuji.493@m.kyushu-u.ac.jp (Y.O.); 3 Department of Biological Sciences, Graduate School of Science and Technology, Kumamoto University, 2-39-1 Kurokami, Chuo-ku, Kumamoto 860-8555, Japan; 4 Graduate School of Life and Environmental Sciences, Shimane University, 1060 Nishitsugawa-machi, Matsue, Shimane 690-8504, Japan; nagato@life.shimane-u.ac.jp; 5 Education and Research Center for Marine Resources and Environment, Faculty of Fisheries, Kagoshima University, 4-50-20 Shimoarata, Kagoshima 890-0056, Japan; uno@fish.kagoshima-u.ac.jp

**Keywords:** polycyclic aromatic hydrocarbons, *Ligia* spp., supralittoral zone, exposure pathway

## Abstract

Polycyclic aromatic hydrocarbon (PAH) concentrations in wharf roach (*Ligia* spp.), as an environmental indicator, and in environmental components of the intertidal and supralittoral zones were determined, and the PAH exposure pathways in wharf roach were estimated. Wharf roaches, mussels, and environmental media (water, soil and sand, and drifting seaweed) were collected from 12 sites in Japan along coastal areas of the Sea of Japan. PAH concentrations in wharf roaches were higher than those in mussels (median total of 15 PAHs: 48.5 and 39.9 ng/g-dry weight (dw), respectively) except for samples from Ishikawa (wharf roach: 47.9 ng/g-dw; mussel: 132 ng/g-dw). The highest total PAH concentration in wharf roach was from Akita (96.0 ng/g-dw), followed by a sample from Niigata (85.2 ng/g-dw). Diagnostic ratio analysis showed that nearly all PAHs in soil and sand were of petrogenic origin. Based on a correlation analysis of PAH concentrations between wharf roach and the environmental components, wharf roach exposure to three- and four-ring PAHs was likely from food (drifting seaweed) and from soil and sand, whereas exposure to four- and five-ring PAHs was from several environmental components. These findings suggest that the wharf roach can be used to monitor PAH pollution in the supralittoral zone and in the intertidal zone.

## 1. Introduction

Polycyclic aromatic hydrocarbons (PAH) are composed of two or more fused aromatic rings without modification and include more than 100 chemical species. PAHs found in the environment are either of petrogenic origin, such as from natural oil seeps and accidental oil spills [1,2,3], or are of pyrogenic origin, which includes volcanic activity [4], the combustion of fossil fuels [5] and organic matter [6], and automobile exhaust [7]. PAHs are ubiquitous contaminants in the atmosphere [8,9], terrestrial soil [10,11], marine and freshwater sediment [12,13,14], seawater [15], and fresh water [16,17], and they can accumulate in a broad range of wildlife [18,19,20,21]. In addition, PAHs are a common contaminant found in the human body [22,23]. However, field data of PAH pollution in marine invertebrates are limited [24], except in bivalves [2,3,25,26]. Field surveys of PAH contamination in invertebrates in the supralittoral zone are particularly sparse [27].

PAH toxicity has been extensively studied. These compounds are well known for their carcinogenicity [28,29], mutagenicity [30], endocrine-disrupting activity [31], and developmental toxicity [32,33]. Owing to their toxicity and distribution, the United States Environmental Protection Agency has selected 16 representative PAHs as priority compounds for environmental monitoring. The International Agency for Research on Cancer has classified benzo[*a*]pyrene into group 1, indicating that it is “carcinogenic to humans”; dibenz[*a,h*]anthracene is in group 2A, indicating it is “probably carcinogenic to humans”.

Surveying the intertidal and supralittoral zones contributes to understanding the toxicity in nearshore organisms. Furthermore, many contaminants accumulate in this area. Therefore, a wide range of pollutants, such as heavy metals [34], pesticides [35], persistent organic pollutants [36,37], pharmaceuticals [38], antibiotics [39], and PAHs in seawater and sediment have been monitored. These contaminants are continuously deposited in coastal areas, with vertical distributions in the intertidal and supralittoral zones. While the intertidal zone is a common focus, the supralittoral zone is also a site of contamination and should also be monitored.

For this purpose, researchers often sample mussels [19,34,40] to conduct biomonitoring in coastal areas. However, mussels may not be appropriate or accessible for environmental biomonitoring in the supralittoral zone. To address this, our research group has identified the wharf roach (*Ligia* spp.) as a suitable environmental indicator for biomonitoring in the supralittoral zone. The wharf roach has a global distribution [41]. It has a relatively small habitat area [42] and is omnivorous [43]; therefore, it is exposed to environmental pollutants because it feeds on land above the tidal line [44]. Environmental pollutants such as radiocesium and radiosilver from the Fukushima Daiichi Nuclear Power Plant [45], tributyltin, dibutyltin [44], and PAHs and alkylated PAHs [46] have been assessed by sampling wharf roaches in the supralittoral zone. Although appropriate as a novel environmental indicator of PAHs in the supralittoral zone, wharf roaches live in the boundary region between the marine and terrestrial environments, and their exposure routes to PAH compounds are unclear.

In this study, the correlations between PAH concentrations in wharf roaches and in environmental components were investigated using field samples, and the exposure pathways by which wharf roaches are exposed to PAHs are estimated. Wharf roaches, mussels, seawater, soil and sand, and drifting seaweed were collected at 12 sampling sites in Japan along coastal areas around the Sea of Japan. These sites were chosen because transboundary PAH air pollution from the continent has been reported in this area [8].

## 2. Materials and Methods

### 2.1. Chemicals and Reagents

A PAH standard mixture containing the following 16 PAHs was purchased from Supelco (Bellefonte, PA, USA): naphthalene (Nap); acenaphthylene (Acy); acenaphthene (Ace); fluorene (Flu); phenanthrene (Phe); anthracene (Ant); fluoranthene (Flut); pyrene (Pyr); benzo[*a*]anthracene (BaA); chrysene (Chr); benzo[*b*]fluoranthene (BbF); benzo[*k*]fluoranthene (BkF); benzo[*a*]pyrene (BaP); benzo[*g,h,i*]perylene (BP); dibenzo[*a,h*]anthracene (DA); and indeno[1,2,3-*cd*]pyrene (IP). The following five deuterium isotopes of PAHs were purchased from Wako Pure Chemical Industries, Ltd. (Osaka, Japan): D_8_-Nap; D_10_-Ace; D_10_-Phe; D_10_-Pyr; and D_12_-BaP. All organic solvents were of pesticide residue and polychlorinated biphenyl test grade. Other chemicals and ultrapure water were of analytical grade.

### 2.2. Sampling

**Figure 1:**
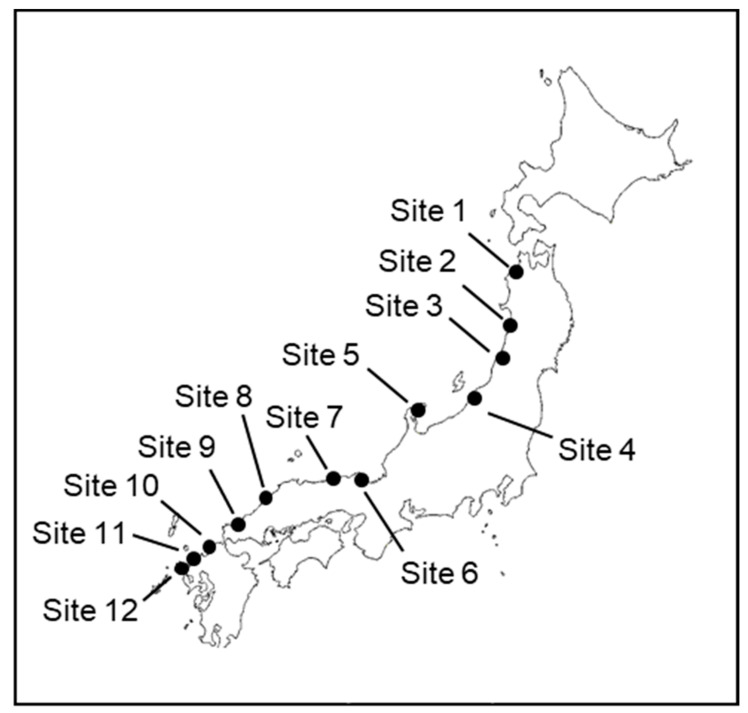
Locations of the 12 sampling sites.

Wharf roaches (*Ligia* spp.) were collected in September 2018 from 12 sampling sites in Japan located along coastal areas of the Sea of Japan (Figure 1, Table 1). Wharf roaches were collected by hand and using tools.

Mussels (*Mytilus* spp.), seawater, soil and sand, and drifting seaweed were also collected. The sampling sites were located in the habitable zone for both wharf roaches and mussels and were suitable for sampling seawater, soil and sand, and drifting seaweed. Mussels were collected from the shore area by hand or using a metal spatula. We collected four liters of seawater from the water surface at each location using brown glass bottles. After collection, the seawater samples were spiked with PAH internal standard mixture and extracted on-site using an Empore C_18_ extraction disc (3M, Maplewood, MN, USA). Soil and sand and drifting seaweed samples were randomly collected from the supralittoral zone near the wharf roach sampling location using a metal shovel or tweezers. All of the samples were immediately stored at 4 °C and sent to the laboratory where they were kept at −30 °C. Debris was frequently observed at the sampling sites (supplementary materials: Figure S1).

### 2.3. PAH Analysis

The analytical methods were conducted as in Ito et al. [19] and Honda et al. [46], with slight modifications. Briefly, the samples (wharf roaches, mussels, drifting seaweed, and soil and sand) were placed in a glass container and freeze-dried for 48 h (FDU-1200; Eyela, Tokyo, Japan). After freeze-drying, 5–10 wharf roach samples were pooled and homogenized (whole body), 2–5 mussels were pooled and homogenized (whole soft tissue), and approximately 1 g of drifting seaweed was pooled and homogenized. Large-grain sand, pebbles, and other foreign matter were removed from the soil and sand samples to produce a roughly uniform grain size. Approximately 0.3 g of wharf roach, mussel, or drifting seaweed and 1 g of soil and sand was weighed and placed into a glass tube. After spiking with the internal standard mixture (20 ng of D_8_-Nap, 4 ng each of D_10_-Ace, D_10_-Phe, D_10_-Pyr, and D_12_-BaP), PAHs were extracted twice with 20 mL of dichloromethane/hexane (50%:50%, *v/v*). The extracts were saponified with 6 mL of 1 M potassium hydroxide in ethanol at 90 °C for 1 h. After saponification, the samples were extracted with hexane. The extracts were washed with ultrapure water and dehydrated with anhydrous sodium sulfate. The dehydrated samples were purified with silica gel (3% water content, *v/v*) and eluted with hexane and 3% acetone in hexane (*v/v*). The eluates were concentrated to 0.2 mL with N_2_ gas and transferred into gas chromatography (GC) vials for analysis. Extraction of PAHs from the C_18_ Empore discs was performed by cutting the disc into pieces and extracting it similarly to the other samples. The uncertainty of single measurements of individual samples was alleviated by sample pooling.

PAHs were quantified using a gas chromatograph (8890 GC system; Agilent, Santa Clara, CA, USA) equipped with a mass spectrometer (5977B GC/MSD; Agilent). Target analytes were separated using a DB-5ms column (0.25 mm i.d. × 30 m length, film thickness 0.25 μm; Agilent). The target analytes were quantified in selected ion monitoring mode. Details of the GC and mass spectrometry parameters are provided in Table S1. The monitored mass of target analytes, the internal standards, and the approximate retention times are provided in Table S2.

### 2.4. Quality Assurance and Quality Control

A method blank and a matrix-spiked sample were analyzed with each batch of 10 samples. Method blanks contained PAH concentrations that ranged from less than the limit of detection (LOD) to 2.79 ng/mL. Matrix effects were corrected using the isotope dilution method of quantification and the retention time. The recovery of PAHs in spiked samples (spiked content: 2–40 ng) ranged from 84.2 (Flu) to 118% (BaA). The instrumental LODs of each analyte ranged from 0.20 to 9.34 ng/mL. Because of the high background and relatively low recovery, Nap was excluded from the analysis.

### 2.5. Statistical Analysis

PAHs with concentrations below the LOD were substituted with the value of LOD/2 for the statistical analysis. R software (ver. 4.0.2, R Development Core Team) was used to conduct the statistical analysis, and the significance level was set at *p* ≤ 0.05. As a non-parametric statistical test, Spearman’s rank–order correlation coefficient was used to examine significant correlations of PAH concentrations among samples. Principal component analysis (PCA) was used to discern whether there were differences between the various sample types. The PCA was performed using SPSS statistics software (ver. 25; IBM, Armonk, NY, USA). Three factors retained based on the scree plot. PCA scores and loading plots were constructed using Microsoft Excel.

## 3. Results and Discussion

### 3.1. PAH Concentrations in Organisms

**Figure 2:**
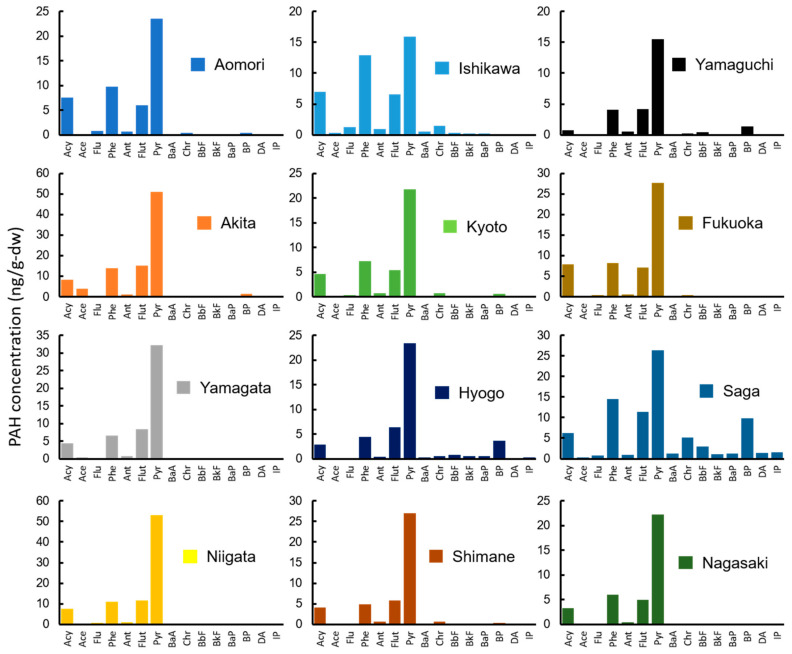
The concentrations of 15 PAHs in wharf roaches (ng/g-dw) collected from 12 sampling sites.

In wharf roach, the highest concentration of the total of 15 PAHs (ΣPAH) was detected in the sample from Akita (96.0 ng/g-dry weight (dw); Figure 2, Table 2), followed by samples from Niigata, Saga, Yamagata, Fukuoka, Aomori, Ishikawa, Shimane, Hyogo, Kyoto, Nagasaki, and Yamaguchi. The detected ΣPAH concentrations (median: 48.5 ng/g-dw, detected range: 26.9 to 96.0 ng/g-dw) were similar to previously reported concentrations, except for samples that were previously collected in highly polluted areas (median: 47.0 ng/g-dw, detected range: 28.6 to 72.2 ng/g-dw) [46]. The PAH composition was largely consistent among the 12 sampling sites (Figure 2). The predominant PAH species was Pyr (median: 24.9 ng/g-dw), followed by Phe, Flut, and Acy (median: 7.71, 6.44, and 5.40 ng/g-dw, respectively). In a previous study, the dominant PAH species were Phe, followed by Ace and Pyr [46]. Thus, the current results differ from previous observations. The composition differences reflect differences in pollution among the sampling sites and differences in contamination sources between sampling seasons (current study: summer, previous study: fall–winter; [47]). High molecular weight PAHs (HMW-PAHs) (i.e., five- and six-ring PAHs) were detected at lower concentrations than low molecular weight PAHs (LMW-PAHs) (i.e., three- and four-ring PAHs), which were the most common. Three-ring PAHs were contributed at 30% and four-ring PAHs at 68% of ΣPAH concentration. It is generally believed that HMW-PAHs do not highly accumulate in organisms because of a lower intake efficiency [48].

**Figure 3:**
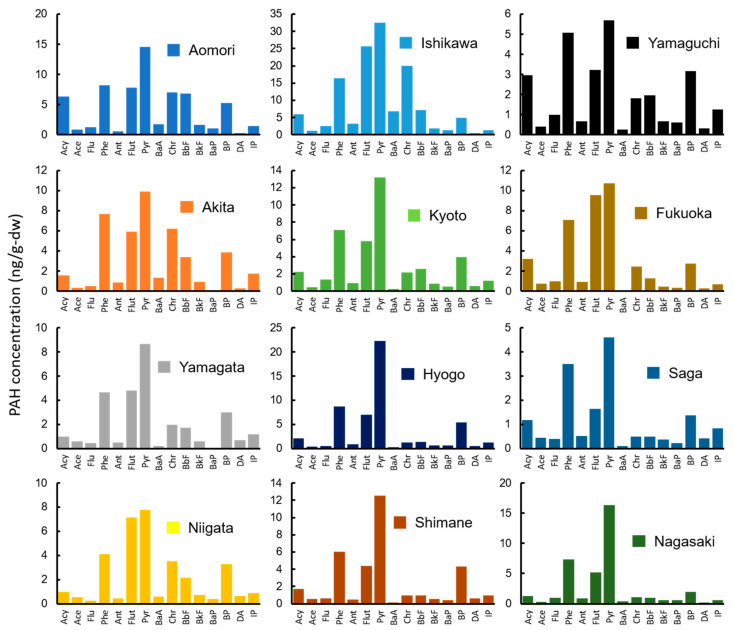
Concentrations of 15 PAHs in mussels (ng/g-dw) collected from 12 sampling sites.

A consistent trend of high Pyr concentrations (median: 11.6 ng/g-dw) was observed in mussel tissue. The highest ΣPAH concentration was detected in a sample from Ishikawa (132 ng/g-dw; Figure 3), followed by samples from Aomori, Hyogo, Akita, Kyoto, Fukuoka, Nagasaki, Shimane, Niigata, Yamagata, Yamaguchi, and Saga. The detected range of ΣPAH among the 12 sampling sites was 16.6 to 132 ng/g-dw. This finding is similar to that of previous studies; 16 PAHs, 87.3–361 ng/g-dw [19]; 18 PAHs, 15.2 to 527 ng/g-dw (both values were converted from initial reported values of 2.6 to 90 ng/g-ww by using an 82.9% moisture content) [40]. Of the 15 PAHs, Pyr was the predominant species (median: 11.6 ng/g-dw), followed by Phe and Flut (7.10 and 5.83 ng/g-dw, respectively). Additionally, the concentrations of HMW-PAHs were greater in mussels than in wharf roaches. The relative standard deviation of ΣPAH among the 12 sampling sites was larger for mussels than for wharf roaches (0.63 and 0.39, respectively). These differences reflect differences in the PAH pollution in the supralittoral and intertidal zones and suggest that wharf roaches and mussels differ in their exposure pathways and bioaccumulation capability.

### 3.2. PAH Concentrations in Environmental Media

**Figure 4:**
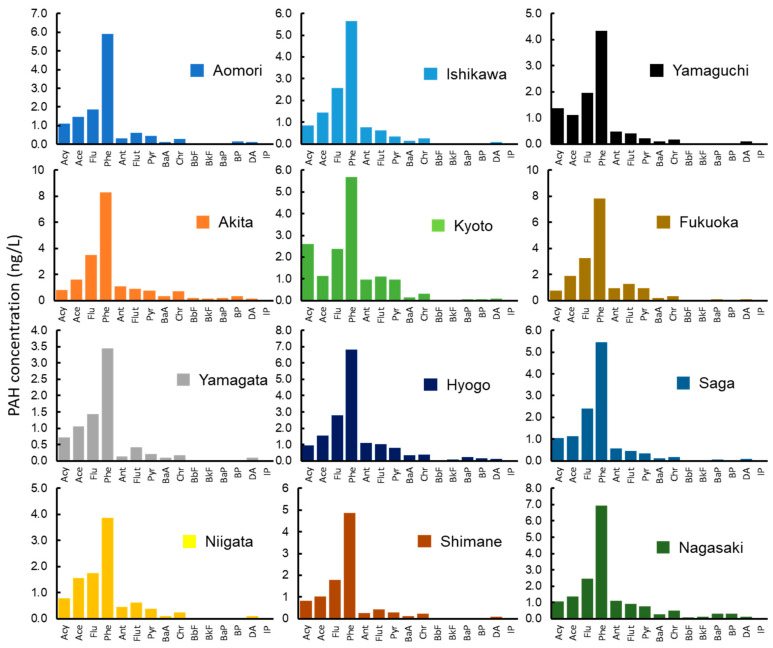
Concentrations of 15 PAHs in seawater (ng/L) collected from 12 sampling sites.

The ΣPAH composition was consistent across seawater samples (Figure 4). The detected range of ΣPAH among the 12 sampling sites was 7.79 to 19.1 ng/L (mean 13.4 ng/L). This finding is similar to that of previous studies; 13 PAHs, 6.83 to 13.81 ng/L (mean 9.4 ng/L) in the Japan Sea [49]; 13 PAHs, 10.9 to 29.7 ng/L (mean 19.6 ng/L) in the Sea of Japan and East Sea [50]. Phe was the predominant species (median: 5.67 ng/L). In general, there was a high contribution of three-ring PAHs to ΣPAH (87%). Comparatively, four-, five-, and six-ring PAHs had very low contributions to ΣPAH (11%, 1.5%, and 0.4%, respectively). The concentrations of LMW-PAHs were higher than those of HMW-PAHs owing to their increased water solubility [47,51].

**Figure 5:**
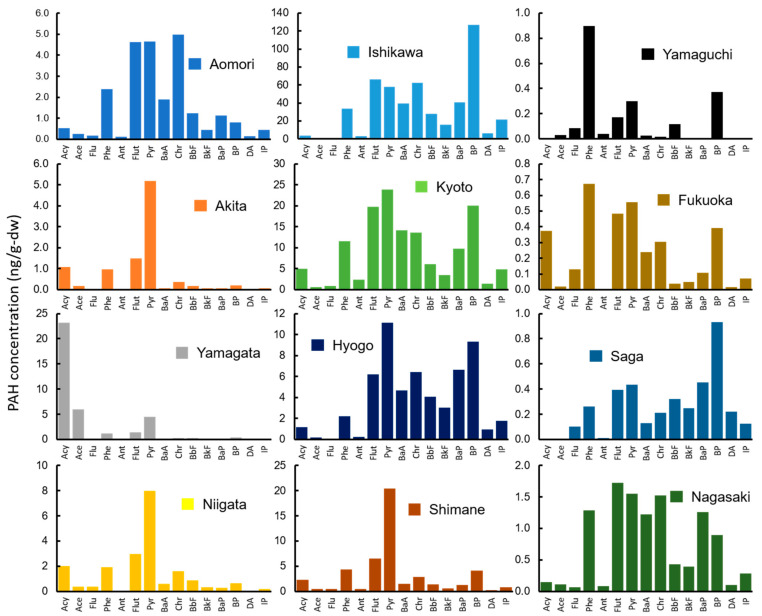
Concentrations of 15 PAHs in soil and sand (ng/g-dw) collected from 12 sampling sites.

The detected range of ΣPAH in soil and sand among the 12 sampling sites was 2.06 to 506 ng/g-dw (median 22.0 ng/g-dw). This finding is lower than that of previous studies; 18 PAHs in sea sediment, 6.40 to 7765 ng/g-dw in Osaka Bay, Japan [12]; 16 PAHs in wharf soil, 842 ± 203 ng/g-dw in Guangdong, China [52]. Phe was the predominant species (median: 5.67 ng/g-dw). At seven sampling sites, that had relatively small particles and a larger soil fraction, the soil and sand samples contained a larger amount of HMW-PAHs than the seawater samples (Aomori, Ishikawa, Kyoto, Hyogo, Fukuoka, Saga, and Nagasaki; Figure 5). The other five sites, which had relatively large sand particles, had PAH compositions similar to that of the seawater samples (Akita, Yamagata, Niigata, and Shimane). The PAH with the highest median concentration detected among the 12 sites was Pyr (4.91 ng/g-dw), followed by Flut and Chr (2.35 and 1.57 ng/g-dw, respectively). There were four-ring PAHs predominant in the ΣPAH at 61%, which was much higher than the percentage of three-ring PAHs (20%). Additionally, five- and six-ring PAHs comprised a much higher percentage of ΣPAH in soil and sand (12.1% and 6.9%, respectively) than in seawater (1.5% and 0.4%, respectively). PAH hydrophobicity is positively correlated with ring number and molecular weight. Higher molecular weight PAHs tend to partition into the particle phase. Therefore, HMW-PAHs were more likely to be found in the soil and sand samples than in the seawater samples. Additionally, the sampling sites of Ishikawa and Kyoto were located in a wharf area, while the other sites were located in beach or rocky areas. Soil samples were collected from a small ditch near a wharf in Ishikawa and Kyoto, and they contained higher concentrations of PAHs (506 and 138 ng/g-dw, respectively) compared with the other 10 sites (median: 15.7 ng/g-dw). This difference was not observed for the other analyzed samples.

**Figure 6:**
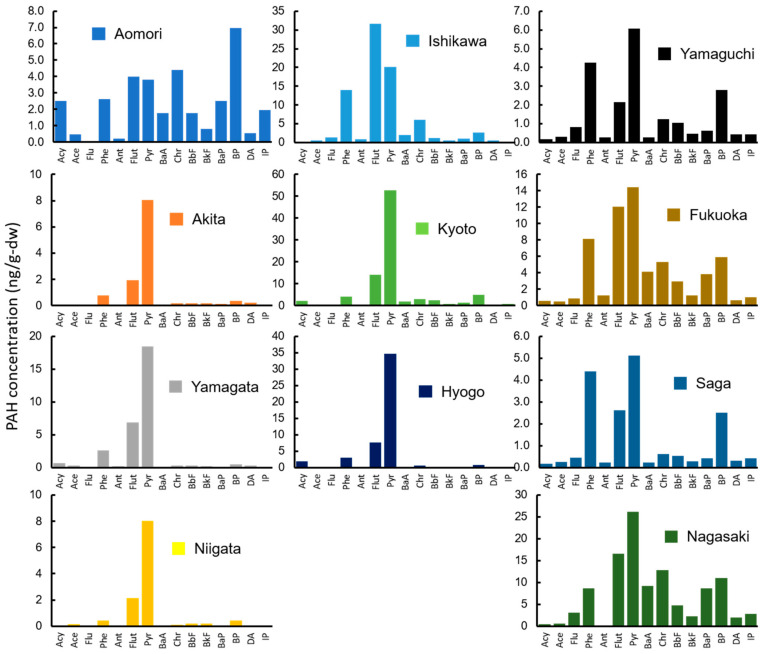
Concentrations of 15 PAHs in drifting seaweed (ng/g-dw) collected from 11 sampling sites. Samples could not be found at the Shimane Prefecture site.

Drifting seaweed is a major food source for wharf roaches. The detected range of ΣPAH in drifting seaweed among the 12 sampling sites was 11.8 to 109 ng/g-dw (mean 47.6 ng/g-dw). This finding is higher than that of previous studies; 16 PAHs in living seaweed, 1.0 to 56.4 ng/g-dw (mean 7.0 ng/g-dw) in Venice, Italy [53]. The PAH composition in drifting seaweed was roughly separated into two groups, with two exceptions (Figure 6) (notably, drifting seaweed samples could not be found at the Shimane site). One group was largely consistent with the PAH trends in seawater (Akita, Yamagata, Niigata, Kyoto, and Shimane). The other group was more similar to the composition found in soil and sand samples (Yamaguchi, Fukuoka, Saga, and Nagasaki). There were two exceptions: Aomori and Ishikawa. As with other environmental media, Pyr was the predominant species (median: 14.4 ng/g-dw). However, Aomori had a higher concentration and percentage of BP (6.95 ng/g-dw, 20%), and Ishikawa had a higher concentration and percentage of Flut (38%). Beyond Pyr, Flut and Phe also had substantial concentrations (6.85 and 4.11 ng/g-dw, respectively). Seaweed can accumulate PAHs from seawater during its lifecycle [53]. Therefore, it is likely that these PAHs were transferred from the seawater and nearby soil and sand into the seaweed. Additionally, aerial deposition on drifting seaweed is a possible exposure source [54].

### 3.3. PCA

**Figure 7:**
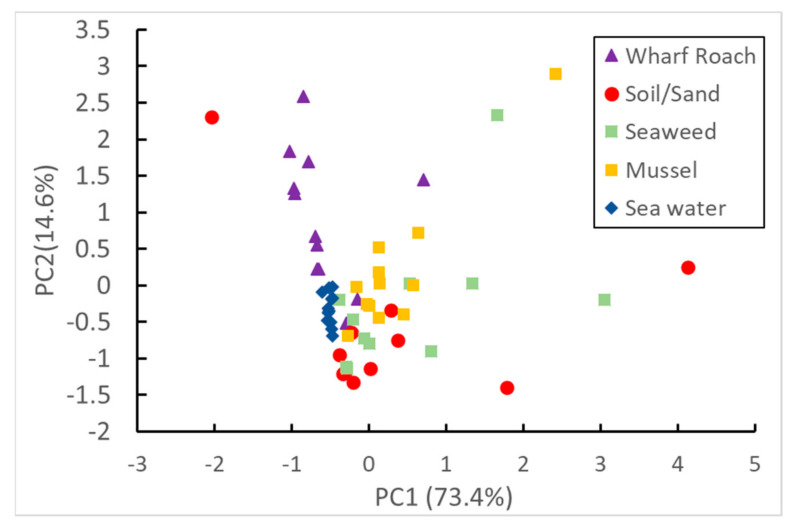
PCA score plot in which the first two PCs account for 88% of the variance.

The PCA confirmed some of the similarities found among the various sample types (Figure 7). In the first principal component (PC) (accounting for 73.4% of the variance), wharf roach and seawater were closely related, whereas soil and sand and mussels formed a related group. The loading plot indicated that the separations in the first PC were largely driven by differences between the three-ring PAHs (i.e., Acy and Ace) and the larger molecular weight PAHs (Figure S2). In general, concentrations of the larger five- and six-ring PAHs were higher in soil and sand, seaweed, and mussels than in wharf roach and seawater (Table S3). By contrast, seawater samples contained greater concentrations of the smaller three-ring PAHs. These differences may account for the separation between these groups in PC1. Compared with the other samples, the relative similarities between soil and sand and mussels were expected given the hydrophobic tendency of PAHs, whereby they partition onto particles or into the high lipid content of mussels. This partitioning also explains the results of the correlation analysis, where four- and five-ring PAHs were particularly well correlated between seawater and wharf roaches. The close association between wharf roaches and seawater supports the idea that the wharf roach can be a useful proxy for assessing surface-layer PAH contamination. However, there appear to be some differences, as seen in PC2, though this accounts for only 14.6% of the variance. Although the loading plot indicated that these separations are caused by differences between the four-ring PAHs and the five and six-ring PAHs, it is not clear why these differences exist.

### 3.4. Identification of Pollution Sources

**Figure 8:**
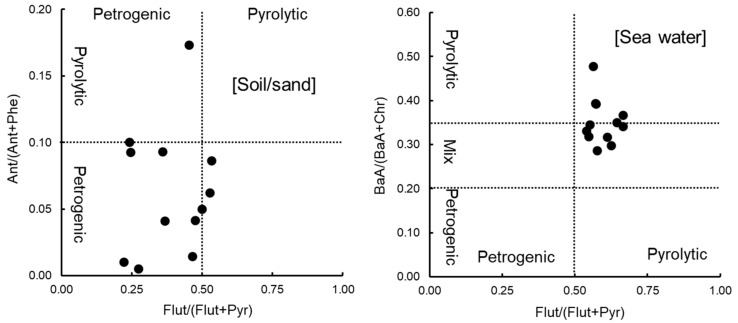
Diagnostic ratios calculated for soil and sand samples and seawater samples.

PAHs are derived from several sources, such as petrogenic, pyrogenic, biogenic, and diagenetic sources [25,55]. PAH pollution in the marine environment is generally thought to be mainly derived from pyrolytic and petrogenic sources. Diagnostic ratios comparing PAH isomers are commonly used to identify pollution sources [2,56,57]. Based on these ratios, petrogenic sources were found to predominate in the soil and sand samples collected at nine of the 12 sampling sites (Figure 8). Pyrolytic sources were detected in samples from three sites (Ishikawa, Kyoto, and Nagasaki), though these associations were weak. Pollution sources for the seawater samples were different from those of the soil and sand samples. The seawater samples likely had a high contribution from pyrolytic sources. Intertidal and supralittoral zones collect floating oil derived from oil contamination from boats or domestic wastewater [5] in the marine environment and are easily polluted by combustion sources located in the surrounding terrestrial area. The ratios found in this study may reflect these phenomena.

### 3.5. Correlation between PAH Concentrations in Wharf Roaches and Environmental Components

Wharf roaches were presumed to be exposed to PAHs through the soil and sand, seawater, and dietary seaweed exposure pathways. Wharf roaches are terrestrial isopods that occupy the supralittoral zone, but they take in seawater through their legs [58]. They feed on drifting seaweed and biofilms [43]. The correlations between PAH ring numbers and organisms and substrates were analyzed. The three-ring PAHs were significantly correlated between wharf roach and drifting seaweed and soil and sand (Spearman’s rank correlation, *p* = 0.01, <0.01; rho = 0.34, 0.51, respectively); the four-ring PAHs were significantly correlated between wharf roach and drifting seaweed, seawater, and soil and sand (*p* = 0.01, <0.01, 0.01; rho = 0.62, 0.59, 0.36, respectively); the five-ring PAHs were significantly negatively correlated between wharf roach and seawater (*p* = 0.02; rho = −0.33). Detected concentrations of five-ring PAHs were relatively low in all analyzed samples. Therefore, it is likely that there was no significant variation in five-ring PAH concentrations to result in a significant correlation between wharf roach and seawater. Because several samples had concentrations of six-ring PAHs that were less than the LOD, correlations for these PAHs could not be analyzed (detection frequency: 80%).

For mussels, the major exposure pathway of LMW-PAHs is generally considered to be water, and that of HMW-PAHs is considered to be particles [59]. This is explained by the hydrophobic properties of PAHs, which make them more likely to partition onto nonpolar particulate surfaces. Mussels continuously filter seawater containing organic matter, which constitutes the majority of its food. For this reason, mussels are liable to accumulate PAHs that are present in the dissolved and particulate phases. The ΣPAH concentration in mussels from Ishikawa (132 ng/g-dw) was the highest among sampling sites, likely because Ishikawa also had the highest soil and sand ΣPAH concentration (506 ng/g-dw).

However, wharf roaches are omnivorous scavengers and are not directly exposed to marine sediment. Food is generally considered the major pathway by which organisms take in PAHs [60]. Surprisingly, the statistical analysis results indicated that LMW-PAHs (i.e., three- and four-ring PAHs) were significantly correlated between wharf roach and food source (i.e., drifting seaweed; rho = 0.34, 0.62). There was also a significant correlation between LMW-PAH concentrations in soil and sand and in wharf roach (rho = 0.51, 0.36). The LMW-PAH composition results indicated that the LMW compounds in wharf roaches had a pattern similar to that in soil and sand (especially in samples from Akita, Niigata, and Shimane; Figure 2 and Figure 5) and to that in drifting seaweed (especially in samples from Akita, Yamagata, Niigata, Kyoto, Hyogo, and Saga; Figure 2 and Figure 6). Some terrestrial isopods accumulate PAHs from contaminated soil [61,62]. In the present study, particles of soil and sand were frequently detected in the intestines of wharf roaches (data not shown). It is possible that wharf roaches directly ingest soil and sand particles to obtain the organic matter adhered to them. This may have been a possible exposure route in the current study. Additionally, van Brummelen et al. [61] reported a negative correlation between biota-to-soil accumulation factors and the PAH K_ow_. Therefore, soil and sand may have contributed less than other routes to the HMW-PAH exposure of wharf roaches. However, the actual contribution ratio of each exposure pathway and the metabolism of PAHs in isopods remain unclear. It is possible that efficient LMW-PAH metabolism lowered their concentrations in the samples, thereby masking a detectable correlation between wharf roach and seawater.

Compared with LMW-PAHs, HMW-PAHs are metabolized more slowly in the body [63,64]; however, HMW-PAHs (more than five rings) are highly hydrophobic and are found less frequently in aquatic samples. Therefore, it is difficult to evaluate the wharf roach exposure pathway to HMW-PAHs. Wharf roaches take in seawater from the ocean surface [58] where there is often floating oil and related hydrophobic substances. Furthermore, the water surface is relatively hydrophobic compared with the underlying water. Therefore, in addition to drifting seaweed and soil and sand, wharf roaches can possibly accumulate a broad range of PAHs via seawater. However, HMW-PAH concentrations in wharf roaches were negatively correlated with those in seawater. This phenomenon may not have indicated a negative association between these two groups, rather, it could have resulted from the relatively low concentrations of five-ring PAHs in the wharf roach and seawater samples. In general, HMW-PAHs are not efficiently taken up and accumulated by organisms because of their high hydrophobicity [63]. Although not significant, in the samples taken from Saga, the relatively high BP concentration in wharf roach may have been related to the high BP concentrations in the soil/sand and drifting seaweed samples. Therefore, it is possible that wharf roaches are exposed to HMW-PAHs through several environmental components; however, no single component has a predominant contribution because of the low intake efficiency.

Bioconcentration, biomagnification, and biota-sediment accumulation have different exposure pathways and contribution ratios that affect the total accumulation. In addition to drifting seaweed, wharf roaches feed on biofilms and other organic matter distributed throughout the supralittoral zone. Additionally, PAH metabolism varies considerably among species. Therefore, it is necessary to expose wharf roaches to several PAHs under laboratory conditions to assess the actual accumulation system and calculate the bioconcentration and biomagnification factors.

## 4. Conclusions

The PAH correlation analysis between wharf roaches and the environmental media (seawater, soil and sand, and drifting seaweed) indicated that exposure to LMW-PAHs occurred mainly via soil and sand and drifting seaweed. HMW-PAH exposure occurred via several environmental components. These findings suggest that wharf roaches can reflect PAH pollution in a broad range of coastal components. To our knowledge, this is the first study estimating PAH exposure pathways for an organism in the supralittoral zone.

There are several limitations to this study. Sorption can occur across many particulate types (e.g., microplastics) and requires further investigation. Additionally, exposure may vary by season and should be further clarified.

## Figures and Tables

**Table 1:** Site locations and sampling date.

Site No.	City, Prefecture	GPS Coordinate	Sampling Date
1	Ajigasawa, Aomori	40.779312, 140.215660	12 September 2018
2	Yurihonjo, Akita	39.501981, 140.045146	13 September 2018
3	Tsuruoka, Yamagata	38.722078, 139.685163	13 September 2018
4	Nagaoka, Niigata	37.646270, 138.764767	14 September 2018
5	Suzu, Ishikawa	37.307115, 137.231492	28 September 2018
6	Miyazu, Kyoto	35.558870, 135.186588	15 September 2018
7	Kami, Hyogo	35.652180, 134.605145	16 September 2018
8	Hamada, Shimane	34.904503, 132.060741	17 September 2018
9	Hagi, Yamaguchi	34.437040, 131.416821	17 September 2018
10	Fukuoka, Fukuoka	33.603976, 130.274923	18 September 2018
11	Karatsu, Saga	33.484003, 129.944348	19 September 2018
12	Hirado, Nagasaki	33.361646, 129.626395	19 September 2018

**Table 2:** Total concentration of 15 PAHs in samples from 12 sampling sites (ng/g-dw or ng/L).

Prefecture	Wharf Roach (ng/g-dw)	Mussel (ng/g-dw)	Soil/Sand (ng/g-dw)	Drifting Seaweed (ng/g-dw)	Sea Water (ng/L)
Aomori	49.2	65.1	23.8	34.4	12.4
Akita	96.0	44.5	9.95	12.4	19.1
Yamagata	53.0	30.1	37.6	31.0	7.79
Niigata	85.2	33.4	20.2	11.8	9.90
Ishikawa	47.9	132	506	82.4	12.8
Kyoto	42.1	43.2	138	89.5	15.6
Hyogo	44.1	53.3	58.1	50.6	16.4
Shimane	44.1	35.4	47.5	na	10.0
Yamaguchi	26.9	29.2	2.06	21.3	10.2
Fukuoka	52.8	41.1	3.45	62.9	17.8
Saga	85.0	16.6	3.85	18.7	11.9
Nagasaki	37.7	38.6	11.1	109	16.4

## Data Availability

Not applicable.

## Supplementary Materials

The following are available online at https://www.mdpi.com/1660-4601/18/2/630/s1, Figure S1: Pollution in the intertidal and supralittoral zones in the Japan coastal area, Figure S2: Loading plot for the first two principal components (accounting for 88% of the variance), Table S1: Gas chromatography (GC) and mass spectrometry (MS) conditions, Table S2: Mass transition and approximate retention time of target analytes and internal standards, Table S3: Total concentration of PAHs grouped by ring number in samples from 12 sampling sites (ng/g-dw or ng/L).
