# Supplementary material for: Correlation between Polycyclic Aromatic Hydrocarbons in Wharf Roach (Ligia spp.) and Environmental Components of the Intertidal and Supralittoral Zone along the Japanese Coast
Source: Int J Environ Res Public Health. 2021 Jan 13;18(2):630. doi: 10.3390/ijerph18020630 (PMC7828494; doi:10.3390/ijerph18020630)
Supplement: Supplementary file 1 [file ijerph-18-00630-s001.pdf]

Table S1. Gas chromatography (GC) and mass spectrometry (MS) conditions.

**GC conditions**	
Column	DB-5ms (0.25 mm i.d. × 30 m length, film thickness 0.25 µm
Injection volume	1 µL
Injection system	Splitless
Injection temp.	270 °C
Carrier gas	He
Gas flow rate	1.2 mL/min
Column temp.	60 °C (1 min hold) - 190 °C up (15°C/min, 3 min hold) - 220 °C up (2 °C/min, 1 min hold) - 300°C up (5 °C/min, 20 min hold)
Interface temp.	270 °C
Ion source temp.	280 °C
Quadrupole temp.	150 °C

Table S2. Mass transition and approximate retention time of target analytes and internal standards.

Target analyte	Quantification ion (m/z)	Confirmation ion (m/z)	Retention time (min)
Acenaphthylene	152	151	9.46
Acenaphthene	154	153	9.75
Fluorene	166	165	10.71
Anthracene	178	176	13.57
Phenanthrene	178	176	13.38
Fluoranthene	202	201	19.35
Pyrene	202	201	20.76
Chrysene	228	226	31.18
Benz[*a*]anthracene	228	226	30.94
Benzo[*b*]fluoranthene	252	253	37.90
Benzo[*k*]fluoranthene	252	253	38.05
Benzo[*a*]pyrene	252	253	39.44
Dibenz[*a,h*]anthracene	278	279	44.44
Indeno[1,2,3-*cd*]pyrene	276	277	45.13
Benzo[*ghi*]perylene	276	277	44.21
D10-Acenaphthene	164	162	9.69
D10-Phenanthrene	188	184	13.30
D10-Pyrene	212	211	20.65
D12-Benzo[*a*]pyrene	264	263	39.32

Table S3. Total concentration of PAHs grouped by ring number in analyzed samples among twelve sampling sites (ng/g-dw or ng/L).

Sample type	Prefecture	3 ring	4 ring	5 ring	6 ring
Wharf roach	Aomori	18.8	29.9	0.07	0.41
	Akita	27.4	66.7	0.16	1.64
	Yamagata	12.1	40.7	0.24	n.d.
	Niigata	20.1	64.8	0.13	0.22
	Ishikawa	22.4	24.4	0.79	0.21
	Kyoto	12.9	28.0	0.40	0.74
	Hyogo	7.79	30.6	1.83	3.90
	Shimane	9.87	33.5	0.33	0.41
	Yamaguchi	5.29	19.9	0.39	1.36
	Fukuoka	17.1	35.2	0.27	0.17
	Saga	22.8	44.0	6.79	11.3
	Nagasaki	10.1	27.3	0.23	0.08
Drifting seaweed	Aomori	5.89	14.0	5.63	8.90
	Akita	1.03	10.1	0.75	0.44
	Yamagata	3.85	25.7	0.97	0.52
	Niigata	0.66	10.3	0.38	0.45
	Ishikawa	16.6	59.8	3.20	2.84
	Kyoto	6.88	71.9	4.98	5.72
	Hyogo	5.82	43.3	0.65	0.77
	Shimane	n.a.	n.a.	n.a.	n.a.
	Yamaguchi	5.77	9.73	2.59	3.24
	Fukuoka	11.4	35.9	8.73	6.87
	Saga	5.53	8.59	1.57	2.96
	Nagasaki	12.9	64.8	17.8	13.9
Sea water	Aomori	10.7	1.43	0.17	0.16
	Akita	15.3	2.73	0.69	0.39
	Yamagata	6.79	0.90	0.11	n.d.
	Niigata	8.42	1.35	0.13	n.d.
	Ishikawa	11.3	1.36	0.15	n.d.
	Kyoto	12.8	2.56	0.21	0.07

	Hyogo	13.2	2.54	0.45	0.16
	Shimane	8.75	1.07	0.18	0.03
	Yamaguchi	9.23	0.86	0.12	n.d.
	Fukuoka	14.7	2.82	0.25	0.06
	Saga	10.6	1.11	0.20	0.04
	Nagasaki	13.0	2.45	0.65	0.32
Soil/sand	Aomori	3.47	16.1	3.59	0.59
	Akita	2.27	7.09	0.50	0.09
	Yamagata	30.5	6.24	0.87	0.02
	Niigata	4.69	13.2	2.10	0.27
	Ishikawa	41.6	226.	211	27.5
	Kyoto	20.5	71.3	39.5	6.29
	Hyogo	3.78	28.5	23.1	2.70
	Shimane	7.88	31.3	7.26	1.01
	Yamaguchi	1.05	0.51	0.49	n.d.
	Fukuoka	1.20	1.58	0.58	0.09
	Saga	0.38	1.17	1.96	0.34
	Nagasaki	1.69	6.02	2.98	0.39

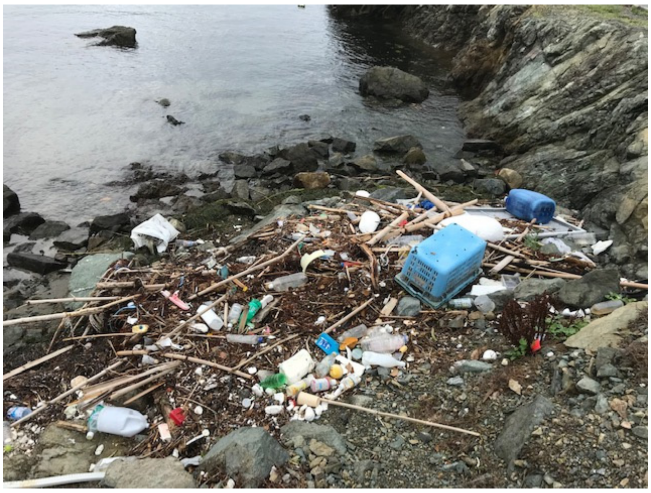

Figure S1. Pollution in the intertidal and supralittoral zones in the coastal Japan area.

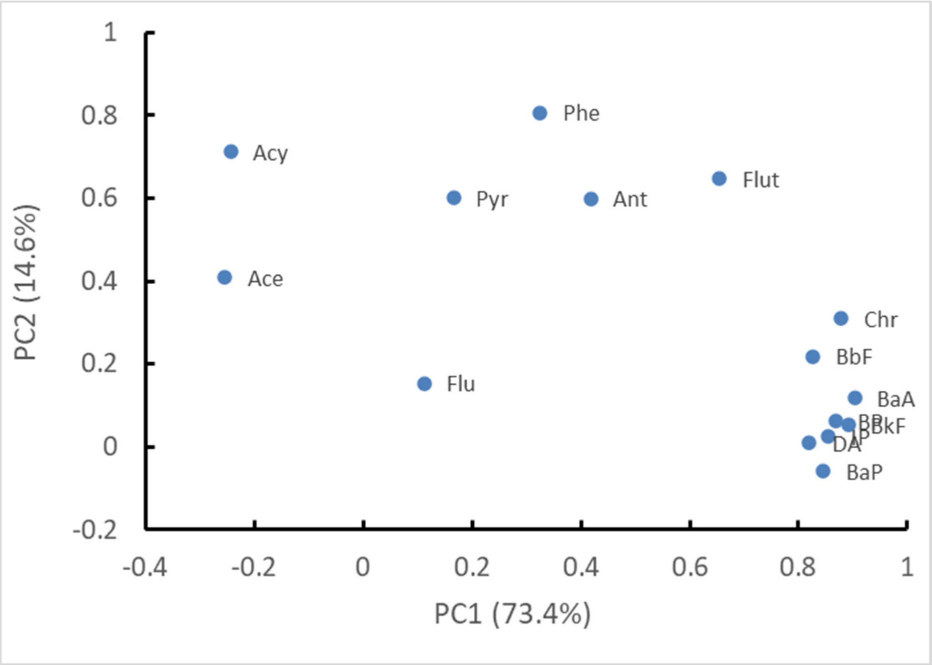

Figure S2. The loadings plot corresponding to the PCA scores plot for the first two PCs (Figure 7), accounting for 88% of the variance.
